# Improving Mechanical Performance of Self-Binding Fiberboards from Untreated Perennial Low-Input Crops by Variation of Particle Size

**DOI:** 10.3390/ma17163982

**Published:** 2024-08-10

**Authors:** Lüders Moll, Alexander Klein, Sören Jannis Heidemann, Georg Völkering, Jessica Rumpf, Ralf Pude

**Affiliations:** 1 Institute of Crop Science and Resource Conservation, Faculty of Agriculture, University of Bonn, Campus Klein-Altendorf, Klein-Altendorf 2, D-53359 Rheinbach, Germanyr.pude@uni-bonn.de (R.P.); 2 Field Lab Campus Klein-Altendorf, Faculty of Agriculture, University of Bonn, Klein-Altendorf 2, D-53359 Rheinbach, Germany

**Keywords:** *Miscanthus*, *Paulownia*, *Picea*, low-input, perennial biomass crop, reaction to fire, biobased, self-binding, fiberboards, sustainable construction

## Abstract

Studies on self-binding hot-pressed fiberboards using agricultural byproducts aim to identify alternatives to scarce wood resources. Particle size and mixture significantly impact strength, although direct comparisons are difficult due to differences in study methods. We evaluated fiberboards made from the two perennial biomass crops *Miscanthus* and *Paulownia* and compared them to *Picea* (spruce), using five distinct particle size blends prepared from milled and sieved particles, respectively. The boards were evaluated for their modulus of elasticity, modulus of rupture, reaction to fire, water absorption, and thickness swelling. All specimens exhibited normal ignitability, as defined by Euroclass E according to EN13501-1. The results indicate that mechanical performance improves with increasing density, which correlates with higher proportions of finer particles. Notably, the finer *Miscanthus* blends and all *Paulownia* samples met the modulus of elasticity requirements of EN 622.

## 1. Introduction

The long-term availability of raw wood from forestry in Germany is expected to decrease from ≈85 × 10^6^ m^3^a^−1^ in 2013 to ≈78 × 10^6^ m^3^a^−1^ in 2052, with ≈44% of the available wood being spruce (*Picea*) [1]. The regional *Picea* stands have been damaged by recent storms and bark beetle infestations leading to high volumes of calamitous wood and quality loss in the absence of preserving measures [2]. As one of the main cost drivers of particleboard production, next to the used binding agents, is the utilized woody biomass, new alternatives like agricultural residues and self-binding fiberboards have to be considered in view of the increasing resource scarcity and competition to strive for increased sustainability in the construction sector [3,4]. Perennial biomass crops are currently being investigated as a sustainable and diversified raw material base for various bio-economic applications [5,6,7]. Of particular interest are crops with high biomass accumulation on marginal sites, such as the C_4_ sweet grass (*Poaceae*) *Miscanthus* with a yield of 10–25 t ha^−1^ a^−1^ [8], or the deciduous tree *Paulownia* [5] (*Paulowniaceae*) which can be cultivated at 5000 trees ha^−1^ and harvested in a 4–6 year cycles for high biomass production [9]. Due to their rapid growth, both cultures are under investigation for bio-energetic [8,9,10] and bio-refining applications [9,11,12], but material applications for *Miscanthus* [13] and *Paulownia* also play an increasingly important role, for example as basis for particle boards [14]. Both biomass plants could thereby supply biomass for materials applications.

Self-binding fiberboards have been researched to obtain new raw materials for the construction industry [4,15,16]. Moreover, it is postulated that the substitution of binding agents could, respectively, reduce the emissions of CO_2_ [16,17] and formaldehyde from wood-based materials [16,18]. Several agricultural by-products such as oil palm biomass [19], olive tree cuttings [17], wheat straw [20], *Cynara cardunculus* [21], *Vitis vinifera* branches [22], and kenaf core [15,23,24,25,26] have been investigated to find possible applications in self-binding fiberboards in various processes.

The core process to manufacture these new materials is the hot pressing of a lignocellulosic feedstock, where thermomechanical deformation induces the adhesive properties of lignins, either by plastic deformation or reaction [16,27]. In addition, the particle size of the biomass is important, as sufficient particle contact is crucial for a good binding [4,16]. To facilitate the binding mechanism, the biomass can be pretreated to soften lignin or release hemicelluloses with different chemical and physicochemical methods [16,28], like fine milling or grinding [28,29], extrusion [30], steam explosion processing (e.g., Masonite process) [29,31,32,33], or chemical reactions in acid or alkaline media or with oxidizing agents, but at the cost of chemical recovery and environmental impacts [4].

The influence of the density of the self-binding boards on their mechanical properties was already observed in some studies: for steam-exploded *Arundo donax* L., the modulus of elasticity (MOE) could be increased from ≈2500 N mm^−2^ [18] at 740 kg m^−3^ to 9552 N mm^−2^ at 1295 kg m^−3^ [34]. Thus, increasing the compaction and density of the boards was found to increase the mechanical strength of the boards [32]. Densities of 810–1306 kg m^−3^ and corresponding MOE values in the range of 1610–6590 N mm^−2^ [31,33] were observed for boards made from steam-exploded *Miscanthus sinensis*. The steam explosion process generally results in acceptable mechanical strength values, but at the expense of energy and special machinery [15]. Furthermore, investigations were conducted to ascertain the efficacy of incorporating lignin [31] or polymeric methylenediphenyl diisocyanate [18] in order to reduce the quantity of steam-exploded fibers, which are associated with high costs.

Self-binding fiberboards can be produced without any pretreatment methods by only milling and pressing [15,24]. In the absence of thermophysical pretreatments, the fiberboards display reduced mechanical and moisture stability values due to the hygroscopic properties of the fibers [4,15]. However, also self-binding boards from finely ground biomass without pretreatment can exhibit higher mechanical strength values when the board densities are increased [19,35]. Particle size is a primary contributor to mechanical bonding properties, as smaller particles can fill voids and act as adhesive by increasing the total contact area [15,16,27,36,37].

The main goal of this study is to examine the influence of finer particles on the mechanical properties of the produced fiberboards. Therefore, different blends with increasing mass share of smaller fractions are prepared, which is an approach that has not been studied yet in the literature. Due to the heterogeneous nature of plants, in general, and the complexity of the precise chemical interactions taking place in the bond formation process [28,38], the comparison of literature values is difficult. This is the first study to directly compare the three different biomasses of calamity wood from *Picea*, and the two perennial biomass crops *Miscanthus* and *Paulownia* in self-binding fiberboards. All three were milled and sieved with common agricultural equipment and processed to self-binding fiberboards without any additional pretreatment. To compare the different products, not only mechanical tests were conducted, but also their reaction to fire and behavior after contact with water (thickness swelling (TS) and water absorption (WA)), since these are important properties for possible future building materials.

## 2. Materials and Methods

### 2.1. Biomasses

**Figure 1:**
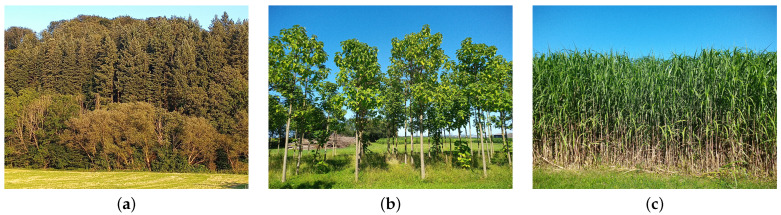
Photographic depiction of of the three different biomass types. (**a**) *Picea* in mixed forest (second level). (**b**) Juvenile *Paulownia* Stand. (**c**) Established *Miscanthus* Stand.

The *Picea* (Figure 1a) wood was chipped from regional calamity wood with a diameter of 8–23 cm. The *Paulownia* biomass was obtained by chipping new shoots (1-year growth) from a plantation maintained at the Campus Klein-Altendorf (Figure 1b). *Miscanthus* × *giganteus* biomass was harvested in April 2022 from a well-established field (Figure 1c) at the Campus Klein-Altendorf (University of Bonn, Rheinbach, Germany) with a forage harvester (Krone Big X 480, Krone, Germany) using a cutting length of 30 mm. All biomasses were crushed using a hammer mill (type BHS 100, F.A. Buschhoff, Germany) equipped with a 1.1 mm sieve.

### 2.2. Fractionation and Mixture of Particle Size Blends

Particle fractions for the mixtures were obtained by sieving on a vibrating sieve (ASM 100, S & F). The control of each biomass was determined by utilizing the full throughput of a 0.5 mm square sieve mesh (V0). Five mixtures were prepared from two fractions (0.25–0.5 mm (V1); 0.25 mm (V5)) in varying rations, as shown in the Table 1.

### 2.3. Hot-Pressing of Fiberboards

**Figure 2:**
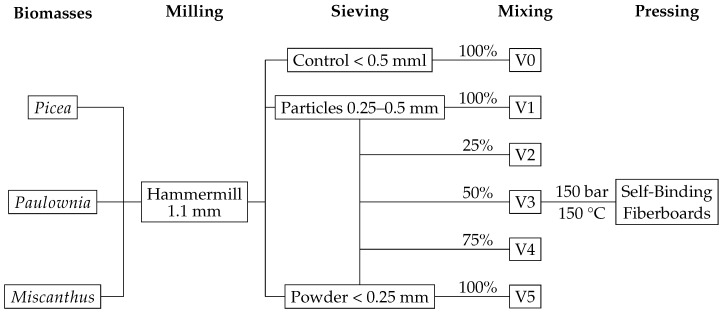
Schematic representation of the sequential steps involved in the production of a variant set, with a particular emphasis on the self-binding fiberboard acquisition process. The different particle size variants are composed of: V0 = native distribution < 0.5 mm; and decreasing particle size from V1 = 0.25–0.5 mm (100%) to V5 = < 0.25 mm (100%) in 25% intervals.

**Figure 3:**
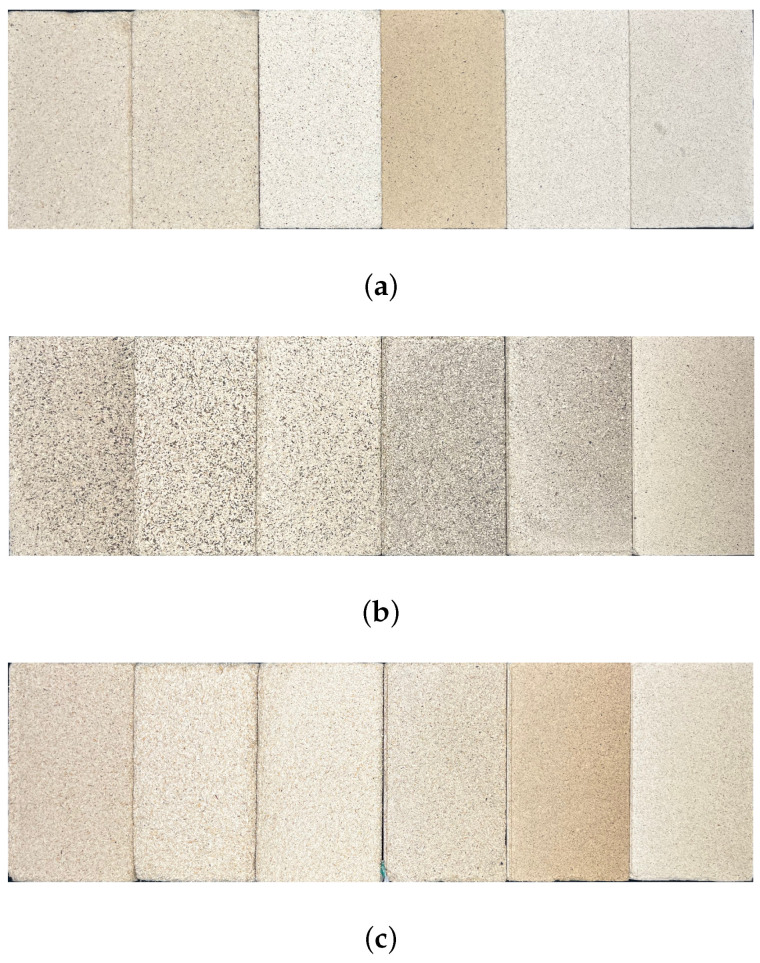
Self-binding fiberboards of particle sizes V0 (left) to V5 (right) from the three different biomasses (**a**) *Picea*, (**b**) *Paulownia*, and (**c**) *Miscanthus*.

The self-binding fiberboards were produced according to the scheme in Figure 2. The powder was hand-formed into a dry mat in a 30 × 30 cm frame and compressed by a plunger in a hot press (Wickert & Söhne, Landau, Germany) at a single step at 150 bar. The core temperature in the mat was monitored with an inserted thermal wire for heat conduction and held at 150 °C for 15 min. The boards were cooled under pressure to 80 °C to prevent explosive vapor release from moisture formed in the boards. Each self-binding fiberboard (Figure 3) variant was manufactured in four replicates.

### 2.4. Mechanical Test

The bending strength of the fiberboards was determined using a Hess universal testing machine (HMN10, Hess MBV GmbH, Sonsbeck, Germany) with a 3-point bending test. The tests were performed after conditioning samples of the dimensions 250 × 50 mm at room temperature in approximation of DIN EN 310 [39] and compared to EN 622-2 [40] as requirements for hard boards in load-bearing dry applications (Tpye HB.LA). The raw density of the boards was determined on the mechanical specimen prior to testing. The Modulus of Elasticity (MOE) [N mm^−2^] was calculated according to Equation (1):

(1) $$\mathrm{MOE}=\frac{l_{1}^{3}\left(F_{2}-F_{1}\right)}{4bt^{3}\left(a_{2}-a_{1}\right)},$$

where $l_{1}$ is the distance between the cylindrical bearings [mm], $F_{1}$ is 10% of the maximum force [N], $F_{2}$ is 40% of the maximum force [N], *b* is the width of the specimen [mm], *t* is the thickness of the specimen [mm], $a_{1}$ is the distance the piston travels at $F_{1}$ [mm], and $a_{2}$ is the distance traveled by the piston at $F_{2}$ [mm].

The modulus of rupture (MOR) [N mm^−2^] can be calculated according to Equation (2)

(2) $$\mathrm{MOR}=\frac{3F_{max}l_{1}}{2bt^{2}},$$

where $F_{max}$ is the maximum force required to break the sample [N].

### 2.5. Reaction to Fire

Reaction to fire tests have been performed on 250 × 90 mm samples, which were exposed to a defined propane flame of 20 mm length, held at an angle of 45 °C, for two flame exposure times of 15 s and 30 s in a KBK 917 small combustion chamber (NETZSCH GmbH & Co. Holding KG, Selb, Germany) according to ISO 11925-2 [41]. After the assigned exposure time, the gas flame was removed. The fire tests were evaluated by measuring the height zone damaged by fire where a threshold of 150 mm may not be exceeded for normal ignitability according to the Euroclass system for the classification of construction materials (EN13501-1 [42]).

### 2.6. Thickness Swelling and Water Absorption

Thickness Swelling (TS) and Water Absorption (WA) tests were performed by fully immersing the samples (50 × 50 mm) in 200 mL tap water for 24 h and recording the changes in weight and thickness. The TS was tested after conditioning the samples at room temperature in approximation to the EN 317 [43] test method.

### 2.7. Statistics

Statistical parameters such as mean, standard deviation, one-way analysis of variance (Tukey HSD at α = 0.05), linearity (Mandel test for non-linearity at α = 0.05) and regression were calculated using the open source programming language R.

## 3. Results and Discussion

### 3.1. Mechanical Evaluation

**Figure 4:**
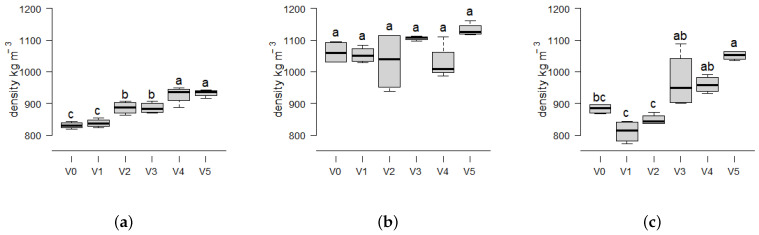
Boxplots showing the density of self-binding fiberboards from the three biomass types *Picea*, *Paulownia* and *Miscanthus* depending on different particle size variants: V0 = native distribution < 0.5 mm; and decreasing particle size from V1 = 0.25–0.5 mm (100%) to V5 = < 0.25 mm (100%) in 25% intervals. Statistical significance is indicated by different letters, representing differences between means based on the Tukey-HSD test at a 95% significance level (*n* = 4).The boxplots consist of the central line representing the median value; the box edges show the 25th percentile (Q1) and 75th percentile (Q3) of the data, with the interquartile range (IQR) as range between Q1 and Q3, representing the middle 50% of the data. The whiskers extend from the edges of the box to the smallest and largest values within 1.5 times the IQR from the quartiles. (**a**) *Picea*. (**b**) *Paulownia*. (**c**) *Miscanthus*.

Figure 4 displays an apparent increase in density with increasing powder proportions from V1 to V5 for *Picea* and *Miscanthus*, which is more pronounced in *Miscanthus* from 883 kg m^−3^ to 1052 kg m^−3^ (Figure 4c) compared to *Picea* from 831 kg m^−3^ to 933 kg m^−3^(Figure 4a). The three biomasses differ in their density development by increasing the proportion of fine particles. *Picea* and *Miscanthus* display an continuous density increase while *Paulownia* shows no statistically significant increase in density. The detailed numeric values for all variants and their respective performance parameters are noted in Table S1 in the supporting information. *Picea* as wood contains a relatively low proportion of parenchyma cells, whereas *Miscanthus* contains a greater proportion of both cortex and parenchyma cells [44,45]. A separation of cortex and parenchyma cells by milling and enrichment of the parenchyma in the powder fraction by sieving may explain the larger increase in density observed in the *Miscanthus* powder. It has been reported that softer cell wall structures, such as parenchyma cells, exhibit greater moldability at a given pressure [28], and thus might influence the density as well as the mechanical properties. The highest overall densities are displayed by *Paulownia* at 1062–1133 kg m^−3^ in Figure 4b illustrating the lack of observed density increase in the tested conditions. This behavior suggests that the softer *Paulownia* [46] particles may be compressed to their limit. This implies that a given density target may be reached with less pressure or particle mixtures containing larger particles when *Paulownia* is compared to *Picea* or *Miscanthus*. The compaction ratio at a given pressure is, therefore, biomass and processing specific.

**Figure 5:**
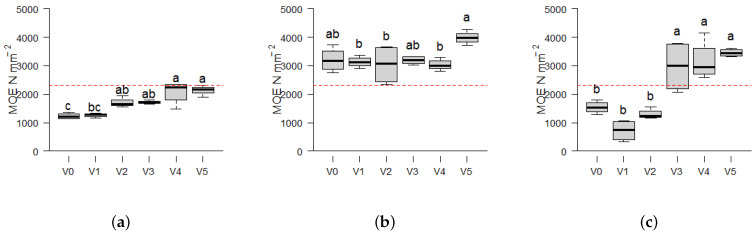
Boxplots showing the Modulus of Elasticity (MOE) of self-binding fiberboards from the three biomass types *Picea*, *Paulownia* and *Miscanthus* depending on different particle size variants: V0 = native distribution < 0.5 mm; and decreasing particle size from V1 = 0.25–0.5 mm (100%) to V5 = < 0.25 mm (100%) in 25% intervals. The red line at 2300 N mm^−2^ marks the threshold for dry load-bearing applications according to EN 622 [40] standards. Statistical significance is indicated by different letters, representing differences between means based on the Tukey-HSD test at a 95% significance level (*n* = 4). The boxplots consist of the central line representing the median value; the box edges show the 25th percentile (Q1) and 75th percentile (Q3) of the data, with the IQR as range between Q1 and Q3, representing the middle 50% of the data. The whiskers extend from the edges of the box to the smallest and largest values within 1.5 times the IQR from the quartiles. (**a**) *Picea*. (**b**) *Paulownia*. (**c**) *Miscanthus*.

By varying the particle sizes and biomasses, binder-free fiberboards with significantly different mechanical strengths can be manufactured. An increase in strength is observed with rising powder content for *Picea* (Figure 5a) and *Miscanthus* (Figure 5c). In general, both exhibit an increase in Modulus of Elasticity (MOE) (Figure 5) and density (Figure 4) as the mass fraction of particles smaller than 0.25 mm increases. The MOE of the *Picea* biomass demonstrates a notable increase by 68 %, from a lowest value in variant V1 of (1265 ± 73) N mm^−2^ to a highest value of (2137 ± 170) N mm^−2^ in variant V5. The greatest mean MOE increase is observed in *Miscanthus*, by 388% from 717 ± 374 N mm^−2^ in variant V1 to 3455 ± 137 N mm^−2^ in variant V5 (Figure 5c). In contrast, *Paulownia* exhibits the highest absolute values with the smallest increase in MOE, as evidenced by the difference between V1 (3124 ± 192 N mm^−2^) and V5 (3886 ± 288 N mm^−2^). This is further supported by the absence of a general increase in board density with increasing dust content, as illustrated in Figure 4b.

The resulting diagrams in Figure 5 demonstrate that certain fiberboards are suitable according to DIN EN 622-2 [40] for dry, load-bearing applications (type HB.LA) with a required MOE of 2300 N mm^−2^. Although density and MOE display an increase from V1 to V5 in *Picea*, the MOE requirements are not met. In contrast, all *Paulownia* variants readily satisfy the MOE requirements, while the boards made from *Miscanthus* reach the threshold of EN 622-2 [40] with at least an amount of 50 % of fine particles (V3–V5). The relationship between compactibility and density to MOE linear relation should be examined to evaluate additional biomasses for their potential use in self-binding fiberboards. The intrinsic compactibility may serve as an indicator of the potential utilization of diverse biomasses in self-binding fiberboards.

In addition to the observed differences in density, variations in lignin content and structure between *Poaceae*, *Conifers* and deciduous trees [47] could further explain the observed differences in mechanical strength of the boards [27,28]. Lignin proportions and structures may differ between biomasses [48,49], particularly in the case of lignin-carbohydrate complexes [50,51]. For instance, the lignin content is estimated to be between 13 and 27% for *Miscanthus* [7], ≈ 22% for *Picea* [52], and 24 and 26% for *Paulownia* [53]. The prevailing theory of effective bonding posits that a chain of factors must converge to achieve bonding [16]. These include particle size to fill voids, geometry to transfer forces, adhesion by physical forces, and adhesion by complex surface chemical reactions [16]. The latter include plastic deformation, or carbohydrate crosslinking [27]. Further research should include the chemical composition of the biomass used and possibly correlate to the density-to-strength linearity. Especially the biomass harvest time or maturation state may influence the chemical composition and thereby the internal bond strength and thus contribute to feedstock quality.

**Figure 6:**
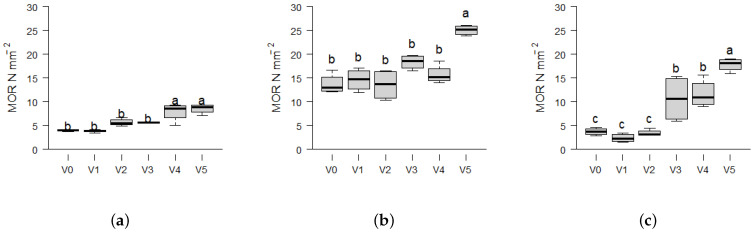
Boxplots showing the Modulus of Rupture (MOR) of self-binding fiberboards from the three biomass types *Picea*, *Paulownia* and *Miscanthus* depending on different particle size variants: V0 = native distribution < 0.5 mm; and decreasing particle size from V1 = 0.25–0.5 mm (100%) to V5 = < 0.25 mm (100%) in 25% intervals. Statistical significance is indicated by different letters, representing differences between means based on the Tukey-HSD test at a 95% significance level (*n* = 4). The boxplots consist of the central line representing the median value; the box edges show the 25th percentile (Q1) and 75th percentile (Q3) of the data, with the IQR as range between Q1 and Q3, representing the middle 50% of the data. The whiskers extend from the edges of the box to the smallest and largest values within 1.5 times the IQR from the quartiles. (**a**) *Picea*. (**b**) *Paulownia*. (**c**) *Miscanthus*.

The Modulus of Rupture (MOR) values increase in a similar manner like the MOE with increasing fineness from V1 to V5, as displayed in Figure 6. For *Picea*, the values increased by 130% from (3.8 ± 0.3) N mm^−2^ for variant V1 to a maximum value of (8.5 ± 1.0) N mm^−2^ for variant V5. In the case of *Miscanthus*, the values increased by 640% from (2.4 ± 0.9) N mm^−2^ for variant V1 to (17.8 ± 1.4) N mm^−2^ for variant V5. *Miscanthus* and *Picea* do not demonstrate a statistically significant increase in MOR at low amounts of powder particles (V1–V2). However, at V3, the density of the *Miscanthus* samples (933 kg m^−3^) is greater than that of *Picea* samples (892 kg m^−3^) and the mechanical differences become more pronounced. *Paulownia* demonstrated the smallest increase in MOR by only 60% accompanied by the highest means. These values ranged from a minimum of (14.6 ± 2.3) N mm^−2^ in variant V1 to a maximum value of (23.2 ± 4.3) N mm^−2^ in variant V5. As with MOE and density, the *Paulownia* biomass thus forms the fiberboards with the highest mechanical strength. The MOR requirement of 30 N mm^−2^ for type HB.LA in load-bearing applications is not met by any variant, but *Paulownia* V5 meets the threshold of 25 N mm^−2^, which is required for the general applications outlined in EN 622-2 [40] (type HB). The existing literature indicates that soft biomasses with lower raw densities, such as *Paulownia* at 317 kg m^−3^ [54], can be compacted more effectively than those with higher intrinsic densities such as *Picea* at 370–571 kg m^−3^ [55]. This results in a higher density of the fiberboards produced [14].

**Figure 7:**
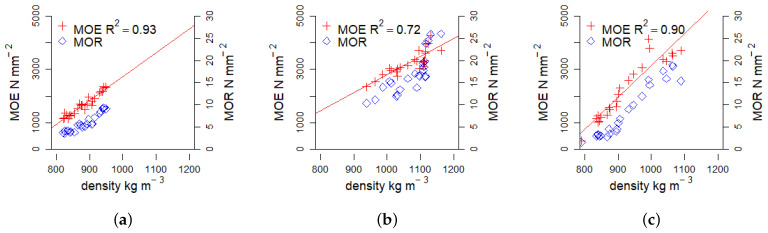
Relationship between Modulus of Elasticity (MOE) and density, as well as Modulus of Rupture (MOR) and density for the three biomass types *Picea*, *Paulownia*, and *Miscanthus*. The coefficient of determination (R^2^) is provided, when the Mandel test favoured linear over quadratic function. (**a**) *Picea*. (**b**) *Paulownia*. (**c**) *Miscanthus*.

Figure 7 and Table 2 shows that there are different linear relationships between the material density and its MOE. This conclusion was reached through the Mandel test for linearity. In contrast, the MOR exhibited a better fit with a quadratic model, thereby rejecting the linear assumption. Particle size effects influence the MOR where elongated particles may provide increased numbers of bonding interfaces compared to fines [16]. The increase in the MOR with the amount of fines and the apparent density indicates that the adhesive effect in self-binding fiberboards without pretreatments is mainly dependent on tight particle packing, which in turn indicates a low adhesive strength or insufficient surface coverage in the chosen conditions.

The density values form distinct biomass-specific clusters. The data points for *Picea* (Figure 7a) are positioned in the first third of the board density range between 800–950 kg m^−3^. For *Paulownia*, they are positioned in the interval of 950–1150 kg m^−3^ (Figure 7b), while *Miscanthus* spans the entire range (Figure 7c). The measured data of density and MOE follow a similar pattern as described for binderless boards from mixtures of coarse particles and flour from Kenaf core [15].

The similar behavior of density, MOE and MOR may allow to use the achieved density of the self-binding fiberboard as a quality assessment parameter to project mechanical performance values and set benchmarks required for specific construction applications. With density as a strength determining factor in this production process, further density increases by pressure or the finer milling of particles could increase the mechanical performance without the need for thermophysical pretreatments.

In order to assess the sustainability of self-binding fiberboards produced via this method, it is essential to conduct a life cycle assessment. Accordingly, the biomass feedstock supply process must be documented in accordance with the requisite energy input for comminution and mass streams subsequent to milling and fractionation. Moreover, the overall production process must be balanced.

From the linearity of density to strength properties, it may be possible to establish reasonable limits for a given material and define its applicable use cases such as materials with a focus on high mechanical performance or materials with lower densities for insulation purposes.

### 3.2. Thickness Swelling and Water Absorption

**Figure 8:**
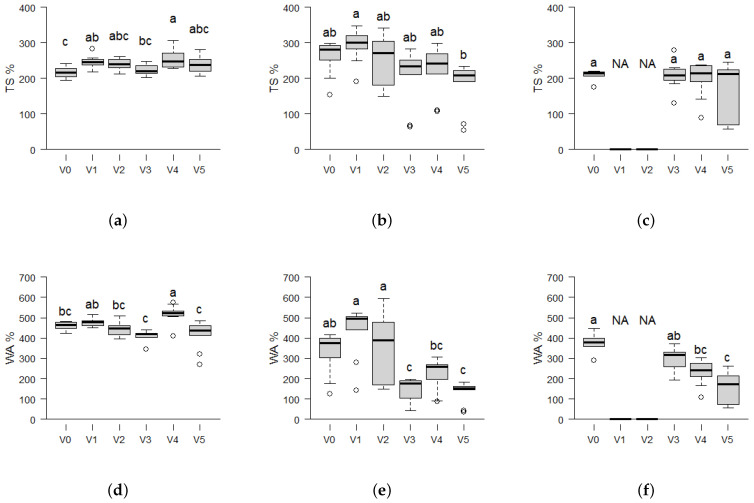
Boxplots showing the Thickness Swelling (TS) and Water Absorption (WA) of self-binding fiberboards from the three biomass types *Picea*, *Paulownia* and *Miscanthus* depending on different particle size variants: V0 = native distribution < 0.5 mm; and decreasing particle size from V1 = 0.25–0.5 mm (100%) to V5 = < 0.25 mm (100%) in 25% intervals. Statistical significance is indicated by different letters, representing differences between means based on the Tukey-HSD test at a 95% significance level (*n* = 10). The boxplots consist of the central line representing the median value; the box edges show the 25th percentile (Q1) and 75th percentile (Q3) of the data, with the IQR as range between Q1 and Q3, representing the middle 50% of the data. The whiskers extend from the edges of the box to the smallest and largest values within 1.5 times the IQR from the quartiles. Outliers are marked by an empty circle. Due to the instability of measurements and significant sample losses (*n* < 5), the variants V1 and V2 for *Miscanthus* have been marked by "NA" and been excluded from the plots and subsequent analysis. (**a**) TS of *Picea*. (**b**) TS of *Paulownia*. (**c**) TS of *Miscanthus*. (**d**) WA of *Picea*. (**e**) WA of *Paulownia*. (**f**) WA of *Miscanthus*.

The Thickness Swelling (TS) displayed in Figure 8a–c ranges from 218 to 254% for *Picea*, 183 to 292% for *Paulownia*, and 175 to 223% for *Miscanthus*. A reduced TS has previously been linked to increased internal bonding strengths in self-binding Kenaf boards by the application of fine powdered biomass [25]. This study indicates weak internal bonds, as evidenced by the fact that only *Paulownia* shows improvement in TS at V5 (183%) compared to the control V0 (260%) in Figure 8b.

To increase the internal bond strength without pretreatments, the adhesive properties may be improved by further softening of the lignin by water addition or increased heat to increase the flowable matrix components as reviewed in literature [16].

The Water Absorption (WA) shown in Figure 8d–f ranges from 407 to 518% for *Picea*, 133 to 428% for *Paulownia* and 159 to 446% for *Miscanthus*. *Miscanthus* exhibits a trend of reduced water absorption with increasing amounts of smaller particle sizes. In general the WA is highest for fiberboards made from *Picea*, while boards from *Paulownia* possess the largest TS, which may be related to the generally lower achieved board densities in *Picea*. This is especially true in the case of the highly densified *Paulownia* boards where V0 displays a TS of 260% at a WA of 327% while *Picea* displays a TS of 217% at a WA of 457%. It was also discovered in literature that TS and WA may exhibit different patterns as water accommodation and thus WA may be proportionally increased at lower board densities due to larger voids, while the TS may remain unaffected. This phenomenon is exemplified by Kenaf at densities of 800 kg m^−3^ and TS values of 7–27% and WA values of 45–80% [25] and self-adhesive insulation boards from felted pine bark at 160–300 kg m^−3^ with TS values below 25% with high WA values of up to 380% [56]. Without pretreatments, water uptake by individual particles will cause high-thickness swelling in dense fiberboards. Future research should, therefore, investigate sustainable routes to decrease the TS by reducing the water uptake of the biomass.

### 3.3. Reaction to Fire

**Figure 9:**
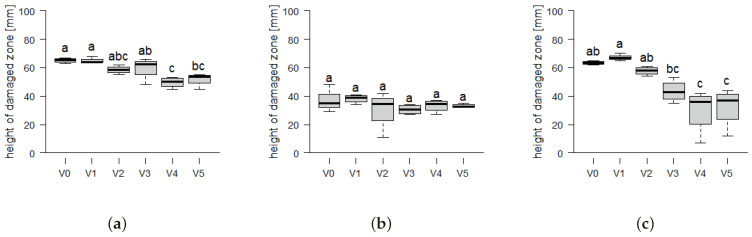
Boxplots showing the height of the damaged zone at 30 s flame exposure of self-binding fiberboards from the three biomass types *Picea*, *Paulownia* and *Miscanthus* depending on different particle size variants: V0 = native distribution < 0.5 mm; and decreasing particle size from V1 = 0.25 – 0.5 mm (100%) to V5 = < 0.25 mm (100%) in 25% intervals. Statistical significance is indicated by different letters, representing differences between means based on the Tukey-HSD test at a 95% significance level (*n* = 4). The boxplots consist of the central line representing the median value; the box edges show the 25th percentile (Q1) and 75th percentile (Q3) of the data, with the IQR as range between Q1 and Q3, representing the middle 50% of the data. The whiskers extend from the edges of the box to the smallest and largest values within 1.5 times the IQR from the quartiles. (**a**) *Picea*. (**b**) *Paulownia*. (**c**) *Miscanthus*.

The fiberboards produced from all three biomasses exhibit a measured height of the damaged zone ranging from 10 to 37 mm after 15 s of surface flaming (Illustrated in the SI in Figure S1). For a period of 30 s, the damaged zone range is increased to 30–67 mm, as illustrated in Figure 9. This results in a Euroclass E rating according to EN 13501-1 [42], as the 150 mm threshold is not exceeded.

The addition of finer particles resulted in a reduction in the damaged zone heights at 15 s flame exposure for *Miscanthus*, with heights decreasing from 29.9 mm in the control to 13.5 mm in V3 (Figure S1). At a prolonged burning time of 30 s exposure *Miscanthus* a damaged zone reduction from 67 mm (V0) to 32 mm (V5) is displayed in Figure 9c. In contrast, the 15 s damaged zone heights for *Paulownia* display the smallest values at 10 mm in V0 (Figure S1), and do not show a statistically significant decrease with increasing powder content at a prolonged flame exposure of 30 s in Figure 9b. The smallest damaged zone heights for *Paulownia* fiberboards could be attributed to its high density ≈ 1000–1100 kg m^−3^ and the inherent low ignitability of *Paulownia* due to its special cell structure [57]. The higher flammability of *Picea* could likewise be a attributed to the lower density of the boards (830–930 kg m^−3^) or the release of flammable volatiles as thermal degradation products from the coniferous wood [57,58]. An increase in density and surface closure of self-binding boards has previously been used to explain reduced damaged zone heights [59]. This effect is consistent with the observed density development of all three biomasses, with *Miscanthus* exhibiting a more pronounced effect than *Picea*, while *Paulownia* demonstrates no significant improvement. In tests with lignosulfonate-bound flax plates with a density of 500 kg m^−3^, flame exposure of 15 s led to damaged zones of 56 mm, which still satisfied Euroclass E [60]. Lower density presumably leads to more air-filled cavities and increased surface areas, thereby increasing the fuel accessibility.

## 4. Conclusions

This study proved the suitability of *Miscanthus*, *Paulownia*, and *Picea* as feedstock material for the production of biobased self-binding fiberboards. No special pretreatment of the biomasses is needed; hammer-milling and screening to obtain the desired particle size is sufficient. Specific targets for mechanical performance may be reached by densification. The apparent density can be increased with the addition of fine powder for some biomasses. Future research could include an even finer powder addition, to examine if further improvement is possible. However, finer grinding consumes more energy, so the determination of the mass use efficiency and energy of comminution might be interesting, to assess the sustainability of the proposed method. Comparing the energy requirements for each route of densification is required to assess sustainability. In addition, the variation in the pressing pressure and its influence on the board density and thus, their mechanical properties, is worth studying.

The biomass *Paulownia* produced the strongest boards with the highest densities, while *Miscanthus* exhibited the most pronounced increase in mechanical properties. To understand the role of finer particles in the binderless fiberboards, the composition of the different mass fractions, especially the accumulation of parenchyma, should be investigated. For other biomasses, the specific compactibility and its density increase by powder addition could be used to assess the general suitability for the process.

The self-binding fiberboard behavior exhibited suboptimal performance after contact with water rendering it unsuitable for applications. According to EN 317 [43], the WA and TS values are too high for all boards under consideration. In ongoing research, the potential of hydrophobic treatments is being explored. The MOE demonstrated norm-satisfying behavior in accordance with the EN 622-2 [40] standard for boards in load-bearing dry applications across all *Paulownia* and specific *Miscanthus* mixtures, while all reaction to fire experiments exhibited satisfactory normal flammability according to the Euroclass system for the classification of construction materials. Thus, the self-binding fiberboards produced in this study seem to be a promising approach paving the way for more sustainability in the construction sector.

## Figures and Tables

**Table 1:** Overview of the preparation of different self-binding fiberboard variants (V1–V5) of *Picea*, *Paulownia* and *Miscanthus*, respectively. The control V0 reflects the native ratio of powder to particles obtained after sieving through a 0.5 mm screen.

Label	Powder <0.25 mm	Particles 0.25–0.5 mm
V0	Control	Control
V1	0%	100%
V2	25%	75%
V3	50%	50%
V4	75%	25%
V5	100%	0%

**Table 2:** Tabulated coefficients describing the linear relationship between density and Modulus of Elasticity (MOE) for the three different biomasses *Picea*, *Paulownia* and *Miscanthus* from this study and reported literature values for self-binding *Kenaf* fiberboards. The $R^{2}$ indicates the goodness of fit for each linear model.

Biomass	Slope [10^−6^ N m kg^−1^]	Intersect [N mm^−2^]	R^2^	Source
*Picea*	9.29	−6507	0.93	Figure 7a
*Paulownia*	6.82	−4020	0.72	Figure 7b
*Miscanthus*	12.53	−9330	0.90	Figure 7c
*Kenaf*	9.26	−4659	0.96	[15]

## Data Availability

The original contributions presented in the study are included in the article/Supplementary Material, further inquiries can be directed to the corresponding authors.

## Supplementary Materials

The following supporting information can be downloaded at: https://www.mdpi.com/article/10.3390/ma17163982/s1, Figure S1: Boxplots showing the height of the damaged zone at 15 s flame exposure of self-binding fiberboards from the three biomass types *Picea*, *Paulownia* and *Miscanthus* depending on different particle size variants: V0 = native distribution < 0.5 mm; and decreasing particle size from V1 = 0.25 – 0.5 mm (100%) to V5 = < 0.25 mm (100%) in 25% intervals. Statistical significance is indicated by different letters, representing differences between means based on the Tukey-HSD test at a 95% significance level (*n* = 4). The boxplots consist of the central line representing the median value; the box edges show the 25th percentile (Q1) and 75th percentile (Q3) of the data, with the IQR as range between Q1 and Q3, representing the middle 50% of the data. The whiskers extend from the edges of the box to the smallest and largest values within 1.5 times the IQR from the quartiles. (**a**) *Picea*, (**b**) *Paulownia*, (**c**) *Miscanthus*; Table S1: Numeric means of all measured properties with respective standard deviations of the three bio-masses *Picea*, *Paulownia*, and *Miscanthus*.
