# Supplementary material for: Improving Mechanical Performance of Self-Binding Fiberboards from Untreated Perennial Low-Input Crops by Variation of Particle Size
Source: Materials (Basel). 2024 Aug 10;17(16):3982. doi: 10.3390/ma17163982 (PMC11355808; doi:10.3390/ma17163982)
Supplement: Supplementary file 1 [file materials-17-03982-s001.zip › materials-3116572-supplementary.pdf]

materials

MDPI

Supplementary Information:

Improving Mechanical Performance of Self-Binding Fiberboards from Untreated Perennial Low-Input Crops by Variation of Particle Size

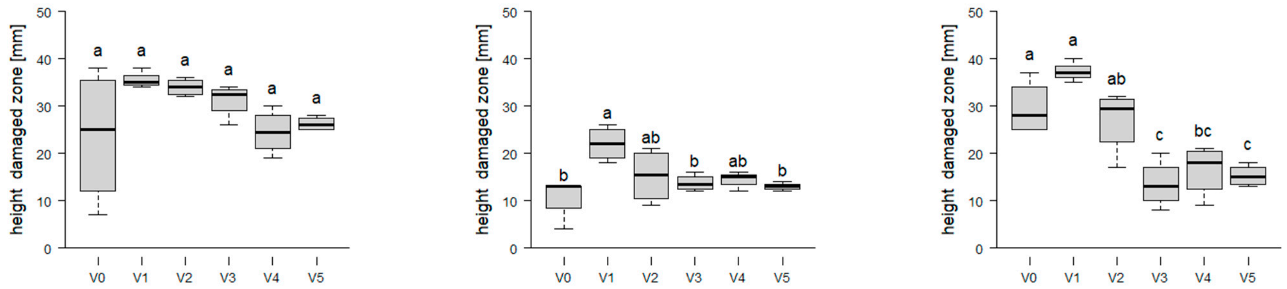

(**a**) (**b**) (**c**)

Figure S1. Boxplots showing the height of the damaged zone at 15 s flame exposure of self-binding fiberboards from (a) *Picea*, (b) *Paulownia*, and (c) *Miscanthus*, depending on different particle size variants: V0 = native distribution < 0.5 mm; and decreasing particle size from V1 = 0.25 – 0.5 mm (100%) to V5 = < 0.25 mm (100%) in 25 % intervals. Statistical significance is indicated by different letters, representing differences between means based on the Tukey-HSD test at a 95 % significance level (n = 4). The boxplots consist of the central line representing the median value; the box edges show the 25th percentile (Q1) and 75th percentile (Q3) of the data, with the IQR as range between Q1 and Q3, representing the middle 50 % of the data. The whiskers extend from the edges of the box to the smallest and largest values within 1.5 times the IQR from the quartiles.

Table S1. Numeric means of all measured properties with respective standard deviations of the three biomasses *Picea*, *Paulownia*, and *Miscanthus*.

Biomass	Label	density	MOE [1]	MOR [2]	TS [3]	WA	RTF 15 s [4]	RTF 30 s [4]
		[kg m^{-3}]	[N mm^{-2}]	[N mm^{-2}]	[%]	[%]	[mm]	[mm]
Picea	V0	831,2 ± 10,2	1230,1 ± 104,5	4,0 ± 0,2	217,6 ± 14,7	457,0 ± 20,8	23,8 ± 14,3	65,3 ± 1,7
	V1	837,5 ± 12,8	1265,1 ± 73,3	3,8 ± 0,2	246,3 ± 16,8	476,6 ± 20,7	35,7 ± 2,1	65,3 ± 2,3
	V2	886,3 ± 19,2	1706,2 ± 171,4	5,7 ± 0,8	239,0 ± 16,4	442,9 ± 34,2	34,0 ± 1,8	58,5 ± 2,9
	V3	886,2 ± 17,3	1712,7 ± 65,5	5,6 ± 0,1	223,1 ± 14,3	406,9 ± 35,2	31,3 ±, 3,6	59,8 ± 8,0
	V4	927,1 ± 28,2	2070,8 ± 407,1	7,9 ± 1,9	253,7 ±25,0	518,3 ± 44,4	24,5 ± 4,7	49,5 ± 3,7
	V5	932,8 ± 12,4	2137,5 ± 170,8	8,6 ± 1,0	238,3 ± 22,3	416,3 ± 69,8	26,3 ± 1,5	51,8 ± 4,6
Paulownia	V0	1061,6 ± 35,8	3197,3 ± 417,3	13,7 ± 2,1	260,1 ± 47,4	327,6 ± 100,7	10,0 ± 5,2	37,3 ± 9,7
	V1	1052,8 ± 25,1	3124,1 ± 192,1	14,6 ± 2,3	292,3 ± 47,9	428,1 ± 131,6	22,0 ± 3,7	38,3 ± 3,1
	V2	1033,1 ± 95,1	3038,5 ± 691,4	13,6 ± 3,2	253,6 ± 67,8	370,5 ± 162,6	15,3 ± 5,7	30,5 ± 13,5
	V3	1106,3 ± 6,7	3189,7 ± 142,5	18,4 ± 1,5	204,2 ± 81,4	143,1 ± 63,6	13,8 ± 1,7	30,5 ± 3,5
	V4	1029,1 ± 54,9	3025,7 ± 196,8	15,7 ± 2,0	227,1 ± 68,1	221,0 ± 76,4	14,5 ± 1,7	33,3 ± 4,5
	V5	1132,7 ± 20,2	3979,8 ± 230,4	25,1 ± 1,1	183,4 ± 65,7	133,5 ± 51,1	13,0 ± 0,8	33,0 ± 1,4
Miscanthus	V0	883,0 ± 14,9	1539,7 ± 214,8	3,7 ± 0,8	206,5 ± 18,4	374,6 ± 56,8	29,5 ± 5,7	63,5 ± 1,3
	V1	811,1 ± 35,4	717,7 ± 374,8	2,4 ± 0,9	223,1 ± 10,5	446,2 ± 44,9	37,3 ± 2,5	67,3 ± 2,5
	V2	849,1 ± 16,6	1291,5 ± 182,3	3,4 ± 0,7	207,0 ± 14,1	353,0 ± 45,9	27,0 ± 6,9	57,8 ± 3,0
	V3	972,1 ± 88,9	2967,0 ± 906,4	10,7 ± 5,0	206,1 ± 37,6	298,9 ± 59,1	13,5 ± 5,0	43,5 ± 7,5
	V4	960,0 ± 26,8	3155,2 ± 689,3	11,6 ± 2,9	200,0 ± 48,7	232,2 ± 60,0	16,5 ± 5,4	30,3 ± 15,8
	V5	1051,9 ± 14,1	3455,1 ± 137,2	17,8 ± 1,4	175,0 ± 76,5	159,3 ± 70,6	15,3 ± 2,2	32,5 ± 14,2

[1] EN 622-2 for type HB.LA MOE requirement ≥ 2300 N mm^{-2}

[2] EN 622-2 for type HB.LA MOR requirement ≥ 30 N mm^{-2}

[3] EN 622-2 for type HB.LA TS requirement ≤ 25 %

[4] EN 13501-1 for Euroclass E ≤ 150 mm
